# Quantitative neurosensory findings, symptoms and signs in young vibration exposed workers

**DOI:** 10.1186/1745-6673-8-8

**Published:** 2013-03-27

**Authors:** Lars Gerhardsson, Lage Burstrom, Mats Hagberg, Ronnie Lundstrom, Tohr Nilsson

**Affiliations:** 1 Occupational and Environmental Medicine, University of Gothenburg, Box 414, Gothenburg, SE-405 30, Sweden; 2 Department of Public Health and Clinical Medicine, Occupational and Environmental Medicine, Umea University, Umea, Sweden; 3 Sundsvall Hospital, Department of Occupational and Environmental Medicine, Sundsvall Hospital, Sundsvall, Sweden

**Keywords:** Vibration exposure, Neurophysiologic effects, Vibrotactile thresholds, Monofilament test

## Abstract

**Background:**

Long-term exposure to hand-held vibrating tools may cause the hand arm vibration syndrome (HAVS) including vibration induced white fingers and sensorineural symptoms. The aim was to study early neurosensory effects by quantitative vibrotactile and monofilament tests in young workers with hand-held vibration exposure.

**Methods:**

This cross-sectional study consisted of 142 young, male machine shop and construction workers with hand-held exposure to vibrating tools. They were compared with 41 non-vibration exposed subjects of the same age-group. All participants passed a structured interview, answered several questionnaires and had a physical examination including the determination of vibrotactile perception thresholds (VPTs) at two frequencies (31.5 and 125 Hz) and Semmes Weinstein’s Monofilament test.

**Results:**

In the vibration exposed group 8% of the workers reported episodes of tingling sensations and 10% numbness in their fingers. Approximately 5–10% of the exposed population displayed abnormal results on monofilament tests. The vibrotactile testing showed significantly increased VPTs for 125 Hz in dig II bilaterally (right hand, p = 0.01; left hand, p = 0.024) in the vibration exposed group.

A multiple regression analysis (VPT - dependent variable; age, height, examiner and five different vibration dose calculations – predictor variables) in dig II bilaterally showed rather low R^2^-values. None of the explanatory variables including five separately calculated vibration doses were included in the models, neither for the total vibration exposed group, nor for the highest exposed quartile.

A logistic multiple regression analysis (result of monofilament testing - dependent variable; age, height, examiner and five vibration dose calculations – predictor variables) of the results of monofilament testing in dig II bilaterally gave a similar outcome. None of the independent variables including five calculated vibration doses were included in the models neither for the total exposed group nor for the highest exposed quartile.

**Conclusion:**

In spite of the fairly short vibration exposure, a tendency to raised VPTs as well as pathologic monofilament test results was observed. Thus, early neurophysiologic symptoms and signs of vibration exposure may appear after short-term exposure also in young workers.

## Background

Vibration exposure may cause a variety of symptoms, depicted as the hand-arm vibration syndrome (HAVS). The symptoms may be of vascular, neural, and muscular origin and may appear as digital vasospasm (vibration white fingers; VWF), sensorineural disturbances [1] and/or as muscular weakness and fatigue. Epidemiologic surveys of vibration exposed workers have found prevalences for sensorineural disorders varying from about two and up to over 80% [2,3]. A decreased tactile sensation has also been noted in e.g. dentists and dental technicians exposed to high-frequency vibration (>1000 Hz) from high-speed hand pieces and ultrasonic scalers [4]. The interindividual susceptibility varies between different subjects and the dose–response relationships are not fully clarified. Some longitudinal studies, however, indicate that symptoms and signs of sensorineural abnormalities have a less favourable prognosis as compared with the vascular symptoms in vibration exposed subjects [3].

Hand-held vibrating tools are commonly used in different occupations. The tools vary in size, weight, acceleration amplitude and frequency. Other factors of importance include vibration impulsiveness, the direction of vibration, the intermittence of exposure, the work methods, the contact force and the posture.

Frequently reported symptoms in vibration exposed workers include loss of sensation, tingling, numbness and paraesthesia in hands and fingers. In long-term exposed workers reduced grip strength and difficulties in performing manipulative tasks are not uncommon [1].

Subclinical changes of sensory dysfunction are not easily diagnosed by clinical examination and a number of psychophysical tests have therefore been developed. Non-painful and non-thermal tactile sensations are caused by mechanical distortion of glabrous skin of four specific cutaneous receptors: 1) The PC channel (mediated by the Pacinian corpuscle) with rapidly adapting nerve fibers with responses in the U-shaped portion between 30 and 500 Hz and with a sensitivity maximum around 250–300 Hz, described as the P channel (Pacinian corpuscle channel) in psychophysical terms. 2) The NP I channel (Non-Pacinian channel I) mediated by Meissner corpuscles with RA fibers, with responses up to approximately 100 Hz and with a maximum sensitivity at 30 Hz. 3) The psychophysical NP II channel (Non-Pacinian channel II), which is probably mediated by paciniform receptors with slowly adapting fibers (SA II). 4) The NP III channel with slowly adapting (SA I) fibers ending in the Merkel-cell neurite complex [5]. In general, temporal and spatial summation has been found in the PC-system. At lower frequencies, however, e.g. at 25 Hz, no temporal or spatial summation was noted. This is the region where the non-Pacinian receptors are most sensitive [5].

The aim of the study was to investigate early neurophysiologic effects, mainly based on monofilament testing and the determination of vibrotactile thresholds, in young workers with hand-held vibration exposure.

## Materials and methods

### Subjects

The original population consisted of 3,000 students who graduated from vocational high-school programs in the northern and south-western parts of Sweden from 2001 to 2003. They received a self-administered questionnaire (SAQ) about vibration exposure and vibration-related symptoms etc. The questionnaire was answered by 1868 young workers, of whom 1,029 volunteered to participate in further studies. This group received the Swedish translation of the VIBRISKS SAQ (http://www.humanvibration.com). Complete questionnaires were returned by 794 workers. Due to reported symptoms and estimated hand-arm vibration exposure, 142 young, healthy male workers (mean age 20.9 y ± SD 1.1 y) were selected for a clinical investigation. In this group, dominant work-sites were auto mechanic shops and construction enterprises. Commonly used tools included screw drivers, grinders, impact drills and jig saws. They were compared with 41 non-vibration exposed male subjects of the same age-group, selected from the catering/restaurant program and part of the group of 794 workers described earlier (mean age 20.7 y ± SD 0.9 y). The study population was first employed during the period 1998 to 2005. All participants passed a structured interview and answered several questionnaires related to e.g. working and medical history, smoking and alcohol consumption, vibration exposure and the year of start, progress and distribution of vibration related symptoms such as white fingers and sensorineural disturbances. To evaluate possible TTS effects, the subjects were also asked whether the neurophysiological symptoms increased after hand-held vibration exposure. A physical examination was performed followed by several tests, e.g. the determination of vibrotactile perception thresholds (VPTs) at two frequencies (31.5 and 125 Hz) and Semmes Weinstein’s Monofilament test. The tests followed the standard ISO 13091–1, Mechanical vibration – Vibrotactile perception thresholds for the assessment of nerve dysfunction – Part 1, Methods of measurement at the fingertips (Geneva, International Organization for Standardization, 2001: 1–21). The subjects were instructed not to work with vibrating tools the day of the measurement and to avoid intake of tobacco and coffee at least one hour before the examination. The study has been approved by the ethical committee of the University of Umea, Sweden.

### Vibrotactile measurements

Measurements of vibrotactile thresholds were performed by delivering sinusoidal vibrations to the pulp of digits II and V, bilaterally (the up-and-down method of limits; von Békésy method), and registering the subjects response, using the HVLab Tactile Vibrometer system (HVLab, United Kingdom). Sinusoidal vibrations at two frequencies (31.5 and 125 Hz) were automatically delivered and transmitted to the finger pulp by a vibration probe (diameter 4 mm) protruding through a circular hole in a rigid plate. These frequencies were chosen to cover the response of Meissner’s corpuscles and Pacini’s corpuscles, respectively [6]. The forearm and the wrist of the participant were supported and the test did not start until the skin temperature of the subject’s forefingers exceeded + 28°C. The test digit applied a force on the surround of 2 N and the contactor applied a force to the digit of 1 N. The magnitude of the vibration was increased until the patient depressed the response button. The vibration magnitude was then decreased until the patient released the response button. Thereafter, the amplitude of the stimulus began to rise again. The rate of change of the vibration amplitude was 3 dB/s and there were six reversals for both frequencies. By connecting a computer, the results for each subject could be compared with an age corrected reference zone. Ear protective devices were used by all participants to mask the noise from outdoor and indoor sources. Several studies have shown good reliability for measurements of vibrotactile thresholds within the 8–500 Hz interval [7]. The method has also shown good validity with intraclass correlation coefficients exceeding 0.94 during long-term follow-ups of patients with diabetic neuropathy [8].

### Monofilament measurements

The Touch Test Sensory Evaluators (Semmes-Weinstein’s Monofilament) provides a non-invasive evaluation of cutaneous sensation levels with results that are valid and repeatable [9,10]. Touch thresholds were assessed at the pulp of digits II and V, bilaterally. The filament was pressed at 90° angle against the skin until it bowed. It was held in place for 1.5 seconds and then removed. For monofilaments with a force in grams from 0.07 g (normal sensation) to 0.4 g (diminished light touch) the stimulus was applied in the same location up to three times to elicit a response. For filaments from 2.0 g (diminished protective sensation) through 300 g (deep pressure sensation only), the stimulus was applied once only. For each finger pulp the testing started with monofilament 0.07 g. If the subject was unable to feel this monofilament, the test continued with 0.2 g, and then if necessary with 2.0 g etc. The subject was sitting with the eyes closed and was told which finger pulp that was tested. A normal response was defined as feeling the thinnest monofilament (0.07 g) on the pulps of digits II and V, bilaterally. A deviating response in this young group of vibration exposed workers was defined as at least one test result of 0.2 g (diminished light touch) or higher on any of the tested four finger pulps. Due to data from questionnaires and anamnesis the examiner knew if the subject tested was vibration exposed or a control. Symptoms and signs related to the vibrotactile perception thresholds and the monofilament testing were related to different indices of vibration exposure. The test-retest reliability of the monofilament procedure is good. In a study in Bangladesh [10], the inter-observer agreement showed a kappa w value of 0.92. In another study of patients with leprosy at a hospital in Nepal, inter-observer weighted kappa values between 0.76 and 0.89 were reported [9].

### Exposure assessment

The exposure to hand-arm vibration was assessed by questionnaire where the respondents answered detailed questions about type of hand-held tools and exposure duration (daily use and time when the exposure started). The vibration magnitude estimates are based on measurements conducted on a sample of hand-held tools among Swedish workers with corresponding work titles and has been documented in a Vibration database (http://www.vibration.db.umu.se/). From this data the number of hours working with hand-held vibration tools were calculated as well as the accumulated vibration dose expressed as exposure time multiplied with the frequency weighted acceleration (a · t) or squared frequency weighted acceleration (a^2^ · t). Moreover, the current 8-hour equivalent frequency weighted acceleration, A(8), according to ISO 5349–1 [11] was calculated. Furthermore, the total vibration dose for both work and leisure time was estimated. Median values and ranges of total hours of vibration exposure (h), a · t weighted total dose, a^2^ · t weighted total dose, current weighted vibration exposure A(8) and total a^2^ · t weighted total dose for work and leisure time for the exposed workers and for the highest exposed quartile, are presented in Table 1.

**Table 1:** Median values and ranges of total hours of vibration exposure (h), a*t weighted total dose, a^2^*t weighted total dose, current weighted vibration exposure A(8) and total a^2^*t weighted total dose for work and leisure time, for all exposed workers and for the highest exposed quartile

**Vibration dose**	**All workers**		**Highest exposed quartile**	
	**Median**	**Ranges**	**Median**	**Ranges**
Total hours exposure (h)	610	5-17550	1925	1490-17550
a*t weighted total dose	1810	6-61315	8785	5365-61315
a^2^*t weighted total dose	8915	10-742545	74445	27005-742545
Current weighted A(8)	1.4	0-5.1	2.8	2.2-5.1
a^2^*t weighted total dose work + leisure	2760	6.1-67215	10210	6585-67215

### Statistics

Parametric statistics were used for comparison of elements that showed a normal distribution (checked by Normal Probability Plots, Levene’s test). For elements with a skewed distribution, nonparametric statistical processing was applied (Mann-Whitney’s U-test). Possible associations between the studied variables were investigated by calculating correlation coefficients (r_s_ = Spearman’s rho). P-values < 0.05 were regarded as statistically significant (2-tailed tests). Multiple linear regression analysis was performed with VPT as the dependent variable and with age (y), height (cm), examiner and calculated vibration doses as predictor variables. Model fits were checked by means of residual analyses [12]. Multiple logistic regression analysis was performed with the outcome of monofilament testing as dependent variable (0 = normal; 1 = at least one deviating monofilament test) and with age (y), height (cm), examiner and calculated vibration doses as predictor variables. The model fits were evaluated by the calculation of Nagelkerke R square. Symptoms and signs of neurophysiologic disturbances were related to different indices of vibration exposure. All calculations were performed with the Statistical Package for the Social Sciences (SPSS v. 18.0) and with SAS, version 9.0.

## Results

### Prevalence of symptoms

The vibration exposed study group consisted of 142 young, male manual workers. Their mean exposure time was 3.1 years (range 1–8 years). In this group 8% of the workers reported tingling sensations, 10% numbness in their fingers and one percent both tingling sensations and numbness in their fingers. These symptoms, however, did not interfere with work or leisure activities. In the reference group there were 41 males. In this group 15% reported tingling sensations and 8% numbness in their fingers, symptoms that did not disturb work or leisure activities. No significant difference was observed when comparing the number of finger phalanges affected with symptoms of tingling sensations or numbness in the two groups.

### Vibrotactile thresholds

The vibrotactile threshold testing showed significantly increased vibration perception thresholds, indicating reduced perception, in the vibration exposed group compared with the referents for digit 2 bilaterally, for the frequency 125 Hz (right hand, p = 0.01; left hand, p = 0.024; Table 2). No significant differences were, however, observed for digit 5 bilaterally or for the VPT testing at 31.5 Hz in digits 2 and 5. The multiple regression analysis (VPT - dependent variable; age, height, examiner and different vibration dose calculations – predictor variables) in digit 2, left hand and right hand, however, produced rather low R^2^-values. None of the five calculated vibration doses were included in the models, neither in the total vibration exposed group, nor in the highest exposed quartile. Moreover, none of the other independent variables gave a significant contribution to the models.

**Table 2:** **Median values and ranges of vibration perception thresholds (m/s**^**2**^**) for 31.5 and 125 Hz in digits 2 and 5, in the right and left hand, in exposed workers (N = 142) and referents (N = 41)**

**Frequency (Hz)**	**Exposed group**		**Reference group**	
	**Digit 2**			
	**Left hand**	**Right hand**	**Left hand**	**Right hand**
31.5 Hz	0.13 (0.03-0.75)	0.15 (0.06-0.81)	0.14 (0.05-0.41)	0.14 (0.05-0.40)
125 Hz	0.17 (0.05-1.16)	0.24 (0.05-0.92)	0.13 (0.04-0.67)	0.16 (0.03-0.96)
Frequency (Hz)	Exposed group		Reference group	
	Digit 5			
	Left hand	Right hand	Left hand	Right hand
31.5 Hz	0.15 (0.03-0.77)	0.17 (0.04-0.79)	0.15 (0.05-0.45)	0.15 (0.07-0.54)
125 Hz	0.24 (0.02-1.45)	0.25 (0.03-1.92)	0.19 (0.06-0.68)	0.21 (0.07-1.15)

### Monofilament thresholds

The number of vibration exposed subjects who displayed abnormal results on monofilament testing was 15 for digit 2 and 8 for digit 5 in the right hand. In the left hand, 14 and 9 subjects, respectively, showed abnormal test results in the corresponding fingers. In the reference group abnormal results for monofilament testing in digits 2 and 5 in the right hand were found in three and two subjects, respectively. Corresponding figures for digits 2 and 5 in the left hand were 5 and 5 subjects, respectively. The logistic multiple regression analysis of digit 2, left hand and right hand, respectively (result of monofilament testing - dependent variable; age, height, examiner and vibration dose calculations – predictor variables) gave low Odds Ratio values. The calculated vibration doses did not give a significant contribution as independent variables in any of the five tested models. No contribution was obtained from any of the other independent variables. This pattern was consistent in the total vibration exposed group as well as in the highest exposed quartile.

## Discussion

Although, the vibration exposure was fairly short a tendency to raised VPTs as well as pathologic monofilament test results was observed. Thus, early neurophysiologic symptoms and signs of vibration exposure may appear after short-term exposure (median exposure time two years) also in young workers. Vibrotactile testing showed a significant increase of the VPTs for 125 Hz in dig II bilaterally, in the exposed workers. This is in accordance with previous studies reporting 125 Hz as the peak sensitivity frequency within the test frequency interval of the instrument [13,14]. The neurophysiologic findings were somewhat more pronounced in the left hand showing bilateral vibration exposure in most workers.

A multiple regression analysis was tested with VPT as dependent variable and with age, length, examiner and five different vibration dose calculations as independent variables. None of the independent variables, however, were included in the models neither in the total exposed group nor in the highest exposed quartile and the corresponding R^2^-values were rather low.

In the total group of vibration exposed subjects, the monofilament testing showed the most significant findings in digit 2, bilaterally. For the monofilament tests, however, none of the five calculated vibration doses contributed significantly to the logistic regression models, nor did the other independent variables.

For digit 2, left hand, one of the exposed workers showed an extremely raised vibration perception threshold, that was around 50% higher, than the highest values among the other workers. If this value was included a marked effect on the results of the analysis was observed with model R^2^-values between 0.3 and 0.5. For digits 2, right hand, and digits 5, bilaterally, this worker however, had vibration perception thresholds that were similar to the other workers. As this high value may have originated from a measurement error it was excluded from further analyses, decreasing the model’s R^2^-values to insignificant levels.

Recent studies have shown a significant exposure-response relationship between VPT and thermal sensory impairment over time and measures of vibration exposure [6]. Thermal perception thresholds (TPTs) are mediated by small myelinated A-δ fibres for cold sensation and by unmyelinated C fibres for warm sensation [15]. VPTs and touch as well as pressure on the other hand are mediated by large myelinated A-β nerve fibres. In our study, the limited exposure time by the works may have been too short to cause substantial effects in the larger myelinated nerve fibers. Similar findings have been reported by Bovenzi et al. [6], in a study of a group of workers exposed to hand-arm vibration.

It is well known that the latency time from the start of vibration exposure to development of early symptoms can vary considerably in different studies. In a study of riveters in the aircraft industry [16], the latency time varied from 0 to 27 years with a median value of 11 years. The average latency time to the onset of symptoms was somewhat shorter, less than five years, in full-time vibration exposed shipyard workers in the US [17]. In our study about 10% of the exposed workers reported tingling sensations and/or numbness in their fingers after a few years of exposure.

Questions about tingling and numbness have shown a high sensitivity for the identification of subjects with sensorineural symptoms from vibration [18]. Neurological disorders can, however, exist without detectable signs and neurological changes can exist without symptoms [19]. The neurological symptoms and signs can reflect a diffuse vibration neuropathy, a carpal tunnel syndrome or a combination of both [20]. None of the workers in our study, however, showed any anamnestic symptoms or signs of a carpal tunnel syndrome.

Earlier studies have shown that neurophysiologic measurements can be a reliable assessment if an initial practice is included as part of the standard administration [21]. Quantitative sensory testing is a valuable tool for the diagnosis of vibration-induced neuropathy [21]. The test of touch sensitivity by Semmes-Weinstein’s monofilament, which was used in this study, is one of the most sensitive predictors of early vibration-induced neurophysiologic effects in hands [22].

Initially, vibration exposure leads to a temporary threshold shift (TTS) of the vibration perception thresholds and to the development of symptoms such as paresthesia and numbness. The TTS increases with the vibration acceleration amplitude and is greater for an exposure frequency of 125 Hz than for 31.5 Hz [14]. The same tendency can be expected to occur for permanent threshold shifts, as observed in our study. Older subjects normally show a decreased sensitivity to vibratory (25 and 100 Hz) and touch stimuli when compared to younger persons. In a recent study by Perry [23] subjects that were 72–73 years old showed a doubling of their detection thresholds as compared to the age-group 65–71 years. In our study, however, the workers were mainly between 20 and 25 years old and thus, an age-dependent effect was not to be expected.

The data from our reference group have been compared with normative data for vibrotactile thresholds, which have been presented by Lindsell and Griffin [24]. The median vibrotactile thresholds in 81 healthy males in their material, where 73% of the subjects were younger than 40 years, were 0.29 m/s^2^ in dig II/III, right hand and 0.27 m/s^2^ in dig II/III, left hand for 125 Hz. These values were higher than the normal values observed in our study. In the study by Lindsell and Griffin [24] also other parameters such as age and outdoor temperature were found to affect the vibrotactile thresholds.

In our study about half of the subjects and referents (N = 83) originated from the northern part of Sweden, and were studied in the area around the city of Umea. The other half of the material (N = 100) originated from the West coast region and was investigated in the city of Goteborg. Previous studies have shown that vibration perception thresholds from 31.5 and 125 Hz measurements are quite similar when comparing centres using similar methods [25]. Thus, the effect of measurement location is often negligible.

The non-occupational vibration exposure among the exposed workers was low as compared with the work-related exposure. The consumption of alcohol and smoking habits were rather similar in the two groups and did not affect the outcome in the multivariate analyses. The exposure to organic solvents was low in both groups and none of the participants had an exposure to potentially neurotoxic substances such as e.g. N-hexane. Workers with symptoms and signs of VWF received advice on improved work practices and preventive measures that could be undertaken to reduce the vibration exposure.

Several indices of vibration exposure have been calculated in this study (Table 1). The vibration estimates were based on measurements conducted on a sample of hand-held tools used among Swedish workers with corresponding work titles. This procedure gives an approximate estimate of the vibration intensity of the tools. However, relatively large variations can be expected due to the level of maintenance of the machine, the type of grinding wheels, type of material and type of work etc.

The estimated time for exposure to hand-held tools is difficult to estimate. An overestimation of the exposure time is not uncommon, sometimes from four [26] and even up to eight times [4]. In the latter study of 10 dental hygienists, the measured use of an ultrasonic scaler was compared with data from subjective estimations based on diaries and interviews. The study showed a large variation regarding the self-estimated exposure time, between as well as within subjects. The self-assessed duration of exposure was overestimated about three times compared to the diary and around eight times compared to the interviews. Accordingly, direct measurements are preferable for adequate risk assessment. In a study by Griffin et al. [27], all measured doses calculated from the unweighted acceleration gave better predictions regarding the risk for developing HAVS as compared with equivalent dose measures using frequency-weighted acceleration according to current standards. Thus, improvements both for the estimation time of vibration exposure and of the frequency weighting may give better possibilities in the future for predicting vibration induced neurophysiologic symptoms.

## Conclusions

In summary, this young cohort has a fairly short cumulative vibration exposure. In spite of this, elevated VPTs as well as abnormal results from monofilament testing were observed in dig II, bilaterally in the vibration exposed group. Thus, early neurophysiologic symptoms and signs may appear after short-term vibration exposure also in young workers. This prospective, longitudinal cohort study gives us a unique opportunity for future investigations. It enables us to detect and evaluate early and late neurophysiologic symptoms and signs in vibration exposed workers in relation to on-going as well as life-long vibration exposure.

## Consent

Written informed consent was obtained from the patients for publication of this report.

## Abbreviations

HAVS: Hand-arm vibration syndrome; VPT: Vibration perception thresholds; VWF: Vibration white fingers

## Competing interests

The authors declare that they have no competing interests.

## Authors’ contributions

LG wrote the manuscript, contributed to the design of the study and to the outcome measurements, participated as examining physician, performed the statistical analyses and the interpretation of the data. MH contributed to the design of the study and to the outcome measurements, participated as examining physician and discussed and contributed to the manuscript. RL contributed to the design of the study and to the outcome measurements, discussed and contributed to the manuscript. LB contributed to the design of the study and to the outcome measurements, discussed and contributed to the manuscript. TN contributed to the design of the study and to the outcome measurements, participated as examining physician and discussed and contributed to the manuscript. All authors have read and approved the final manuscript.
